# Insights into the sonochemical synthesis and properties of salt-free intrinsic plutonium colloids

**DOI:** 10.1038/srep43514

**Published:** 2017-03-03

**Authors:** Elodie Dalodière, Matthieu Virot, Vincent Morosini, Tony Chave, Thomas Dumas, Christoph Hennig, Thierry Wiss, Oliver Dieste Blanco, David K. Shuh, Tolek Tyliszcak, Laurent Venault, Philippe Moisy, Sergey I. Nikitenko

**Affiliations:** 1 Institut de Chimie Séparative de Marcoule, ICSM- UMR5257, CNRS/CEA/UM/ENCSM, Site de Marcoule, BP 17171, 30207 Bagnols-sur-Cèze, France; 2 CEA/DEN/MAR/DRCP, Nuclear Energy Division, Radiochemistry and Process Department, BP17171, 30207 Bagnols-sur-Cèze, France; 3 Helmholtz-Zentrum Dresden-Rossendorf, Institute of Resource Ecology, Bautzner Landstrasse 400, 01328 Dresden, Germany; 4 European Commission, Joint Research Centre (JRC), Institute for Transuranium Elements (ITU), Postfach 2340, 76125 Karlsruhe, Germany; 5 Chemical Sciences Division, Lawrence Berkeley National Laboratory, One Cyclotron Road, Berkeley, CA 94720, USA; 6 Advanced Light Source (ALS), Lawrence Berkeley National Laboratory, One Cyclotron Road, Berkeley, CA 94720, USA

## Abstract

Fundamental knowledge on intrinsic plutonium colloids is important for the prediction of plutonium behaviour in the geosphere and in engineered systems. The first synthetic route to obtain salt-free intrinsic plutonium colloids by ultrasonic treatment of PuO_2_ suspensions in pure water is reported. Kinetics showed that both chemical and mechanical effects of ultrasound contribute to the mechanism of Pu colloid formation. In the first stage, fragmentation of initial PuO_2_ particles provides larger surface contact between cavitation bubbles and solids. Furthermore, hydrogen formed during sonochemical water splitting enables reduction of Pu(IV) to more soluble Pu(III), which then re-oxidizes yielding Pu(IV) colloid. A comparative study of nanostructured PuO_2_ and Pu colloids produced by sonochemical and hydrolytic methods, has been conducted using HRTEM, Pu L_III_-edge XAS, and O K-edge NEXAFS/STXM. Characterization of Pu colloids revealed a correlation between the number of Pu-O and Pu-Pu contacts and the atomic surface-to-volume ratio of the PuO_2_ nanoparticles. NEXAFS indicated that oxygen state in hydrolytic Pu colloid is influenced by hydrolysed Pu(IV) species to a greater extent than in sonochemical PuO_2_ nanoparticles. In general, hydrolytic and sonochemical Pu colloids can be described as core-shell nanoparticles composed of quasi-stoichiometric PuO_2_ cores and hydrolyzed Pu(IV) moieties at the surface shell.

Plutonium is omnipresent in the environment as a result of nuclear weapon tests and nuclear power plant accidents. Colloidal species of Pu(IV) have been shown to play a central role in the speciation of plutonium in several aquatic systems^1^,^2^,^3^,^4^. Recent studies have demonstrated that both intrinsic and pseudo Pu colloids may facilitate the subsurface transport of plutonium^5^,^6^. However, a more comprehensive understanding of Pu colloid properties remains elusive and hinders progress on the development of reliable processes to control their behavior, and thus, effectively manage their environmental impact. The preparation of intrinsic Pu colloids with controlled composition and properties is still a great challenge. In general, colloidal Pu species obtained by hydrolysis of Pu(IV) salts are composed of 2–5 nm PuO_2_-like nanoparticles (NPs) coated by Pu(IV) ions coordinated to water molecules, OH^−^ groups, and anions originating from electrolyte salts^7^,^8^,^9^,^10^. In organic solvents, ~5 nm PuO_2_ nanoparticles obtained by thermolysis of Pu(VI) in a mixture of benzyl ether, oleic acid, and oleylamine was shown to be stabilized by oleate-anions adsorbed at the surface of PuO_2_ NPs^11^. The presence of stabilising solvents and ligands may influence the behaviour of Pu intrinsic colloids such as their adsorption onto mineral surfaces and redox properties just to name a few. Therefore, a reliable and reproducible synthetic route to prepare Pu colloids free from the influence of stabilizing species is highly desirable.

Sonochemistry (chemistry driven by the application of ultrasound) is a well-developed technique to access nanoparticles with controlled properties^12^,^13^. Sonochemistry does not arise from the direct action of ultrasonic waves on molecules but rather from the acoustic cavitation phenomenon within a liquid, which is a set of consequent events: nucleation, growth, and rapid implosive collapse of gas- and vapour-filled microbubbles reaching an acoustic resonance size. Violent implosion of the cavitation bubbles generates transient extreme conditions in the gas phase of the bubble which are responsible for the formation of chemically active species. In principle, each cavitation bubble serves as a plasma chemical microreactor that provides a highly energetic environment at almost room temperature of the bulk solution^14^,^15^. Mechanical effects of ultrasound in heterogeneous solid/liquid systems (acceleration of mass transfer, surface erosion, grain-size reduction etc.) originate from numerous secondary effects from acoustic cavitation, such as acoustic streaming, micro-jetting, splashing, shock waves and shearing forces^16^. In this work, we report for the first time the preparation of stable salt-free Pu intrinsic colloids by the action of ultrasonic waves on PuO_2_ powder in pure water. The comparative study of nanostructured PuO_2_, and hydrolytic and sonochemical Pu colloids, brings new insights into the influence of particle size on the local structure of PuO_2_.

## Results and Discussion

### Kinetics and mechanism of Pu colloid formation

**Figure 1:**
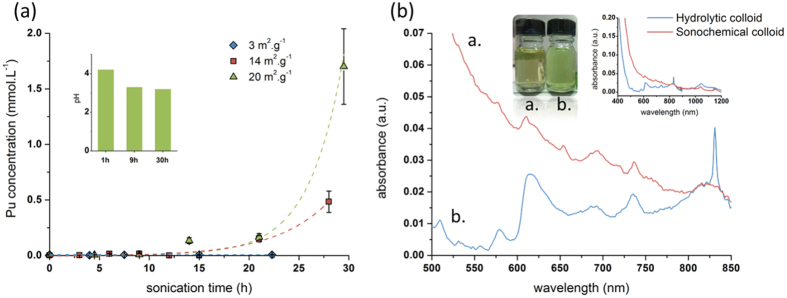
Formation kinetics and Vis-NIR spectra of Pu intrinsic colloids. **(a)** Kinetic curves of sonochemical colloid formation during sonolysis of PuO_2_ in water at 20 kHz (P_ac_ = 0.34 W·mL^−1^) in the presence of 10%CO/Ar gas mixture at 20 °C. Concentration of Pu in colloidal solution was measured by alpha spectroscopy immediately after removal of non-colloidal particles of PuO_2_. Inset shows pH evolution during sonolysis of PuO_2_ (S_BET_ = 20 m^2^∙g^−1^). After 30 h of sonolysis the pH value of solution was 3.3 ± 0.1 for all studied PuO_2_ samples including the experiments in the presence of Ar and Ar/O_2_. **(b)** Vis-NIR absorption spectra of sonochemical Pu colloid, [Pu] = 1.7·10^−3^ M, pH = 3.2 (a.) and hydrolytic Pu colloid, [Pu] = 2.3·10^−3^ M, pH = 2.8 (b.). Insets show a picture of both colloids and the extended Vis-NIR spectra. Vis-NIR spectra were collected after 1 month of sampling.

Prolonged ultrasonic treatment of stoichiometric PuO_2_ suspensions in water sparged with pure Ar or with a 10%CO/Ar gas mixture was found to yield optically clear brown-green colloidal solutions stable for at least 6 months. The yield of Pu colloid obtained under 10%CO/Ar gas mixture was found to be almost 3 times greater than that observed under pure Ar (Table S1). Consequently, most of the sonochemical experiments in this study were performed with 10%CO/Ar gas mixtures. Figure 1a demonstrates that the rate of Pu colloid formation strongly increases with the increase of PuO_2_ specific surface area. In addition, the kinetic curves display an induction period. This observation can be explained by metal oxide particle fragmentation at the initial stage of ultrasonic treatment that leads to an increase of the specific surface area of solids. Scanning electron microscopy (SEM) images of PuO_2_ and ThO_2_ powders before and after ultrasonic treatment shown in Figures S1 and S2 clearly substantiate particle size reduction. This phenomenon has been assigned to the effect of shock waves produced during cavitation bubble collapse in close vicinity to metal oxide particles^17^. Interestingly, the sonication of a PuO_2_ suspension was found to be accompanied by a slight acidification of the solution as illustrated in the inset of Fig. 1a. This observation can be related to Pu(IV) hydrolysis at freshly formed solid surfaces after particle fragmentation.

Figure 1b shows the Vis-NIR absorption spectra of Pu colloids prepared by hydrolysis of Pu(IV) in a homogeneous bulk solution (hydrolytic colloid) and by sonolysis of PuO_2_ suspension in water (sonochemical colloid). The spectral features of hydrolytic colloid are found to be well correlated with the absorption spectra of intrinsic Pu(IV) colloids reported previously^18^,^19^. The sharp absorption peak at 830 nm indicates the presence of Pu(VI) which is formed via Pu(IV) disproportionation in a weakly acid solution^19^:


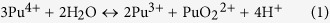


Kinetic data shown in Supplementary Information indicates that formation of hydrolytic Pu colloid should be accompanied by a Pu(IV) disproportionation. On the other hand, in nitric medium Pu(III) undergoes rapid oxidation to Pu(IV). In addition, molar absorption coefficient of Pu(III) (ε = 38 cm^−1^·M^−1^) is low compared to Pu(VI) (ε = 555 cm^−1^·M^−1^)^16^. Consequently, Pu(III) is not observable in Vis-NIR spectrum of the hydrolytic colloid.

Like a hydrolytic colloid, the Vis-NIR spectrum of sonochemical colloid exhibits peaks at 615, 690, 735, and 820 nm (Fig. 1b). On the other hand, the strong Mie scattering observed in the spectrum of sonochemical colloid that spans from the UV to NIR spectral range (see Fig. 1b inset) is most probably explained by larger particles compared to hydrolytic colloid. Pu(VI) absorption is not observable in the spectrum of sonochemical colloid indicating that the mechanism of its formation is different from that of hydrolytic colloid.

Plutonium dioxide is known to be a very unreactive material towards dissolution even in most highly concentrated acids^19^. Therefore, the mechanism of Pu sonochemical colloid formation in pure water is an intriguing question. As mentioned above, the initial slow stage of colloid formation kinetics (Fig. 1a) suggests a strong contribution from ultrasonically–driven particle size reduction in the overall process. Mechanical effects from ultrasound alone, however, are not sufficient to initiate Pu colloid formation. For example, the extended sonication of ThO_2_ (30 h, S_BET_ = 17 m^2^·g^−1^) in pure water does not lead to colloid formation ([Th] < 0.1 ppm, Table S1) despite the pronounced fragmentation of ThO_2_ particles (Figure S2) and slight acidification of sonicated solution (pH = 4.4) similar to PuO_2_. This result is in line with previous study which revealed the absence of Th colloid formation during the prolonged contact of crystalline ThO_2_ with weakly acid aqueous solutions^20^. According to the literature, the pH value of isoelectric point for PuO_2_ and ThO_2_ is equal to 8.6^6^ and 7.5^21^ respectively and both colloids, Pu and Th, would be positively charged in the pH range of 3.3–4.4 studied in this work. In consequence, the striking difference between PuO_2_ and ThO_2_ under ultrasonic treatment in water cannot be attributed to their particle charge. On the other hand, it is well known that in contrast to Pu(IV), Th(IV) does not exhibit redox properties in aqueous solutions. Therefore, one can expect that the mechanism of Pu intrinsic colloid formation involves redox reactions of Pu(IV) triggered by ultrasound in addition to mechanical effects.

The yield of Pu sonochemical colloid (G, μmol) is strongly influenced by the saturating gas. The highest G value is observed for 10%CO/Ar gas mixture (Table S1). In contrast, colloid formation is completely inhibited in the presence of oxygen. These results can be understood in terms of sonochemical reactions occurring in an aqueous medium. In the presence of pure argon, acoustic cavitation causes homolytic splitting of water molecules^14^,^15^:


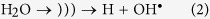



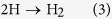



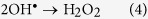


where the symbol “)))” indicates a process triggered by a cavitation event. It is generally recognized that the sites of mutual recombination for H atoms and OH^•^ radicals are different: H atoms recombine inside the bubble, whereas the recombination of OH^•^ radicals occurs mostly at bubble/liquid interface^22^. Carbon monoxide is known to be an effective intrabubble scavenger of OH^•^ radicals leading to the increase of hydrogen yield^22^:


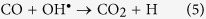


Contrasting to the CO case, H_2_ production during water sonolysis is sharply decreased in the presence of oxygen^23^:


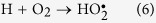



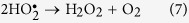


Therefore, one can suggest that *in situ* generated hydrogen is involved in Pu(IV) reduction at the interface of PuO_2_ followed by the solubilisation of transient Pu(III) species:


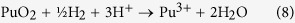


Local heating produced by bubble collapse could be a driving force of this process. Then Pu(III) would be rapidly oxidized at bubble/solution interface yielding Pu(IV). Following this step, intrinsic Pu colloid may be formed according to the following generalized hydrolytic reactions^1^,^2^:


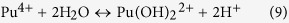



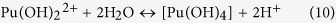


with [Pu(OH)_4_] representing the oxyhydroxide colloidal species Pu_n_O_p_(OH)_4n-2p_(H_2_O)_z_ (0 ≤ p ≤ 2n). Such mechanism presumes low concentration of Pu(IV) in solution. Considering a second-order of Pu(IV) disproportionation upon Pu(IV) concentration, one can conclude that this reaction should not influence colloid formation during PuO_2_ suspension sonolysis. Finally, formation of the sonochemical colloid is assumed to take place at the interface between the collapsing bubbles and PuO_2_ particles. Obviously, the mechanical effects produced by acoustic cavitation would play a significant role in such a mechanism by providing a larger surface area for contact between the bubbles and solids.

### High resolution transmission electron microscopy (HRTEM) study

**Figure 2:**
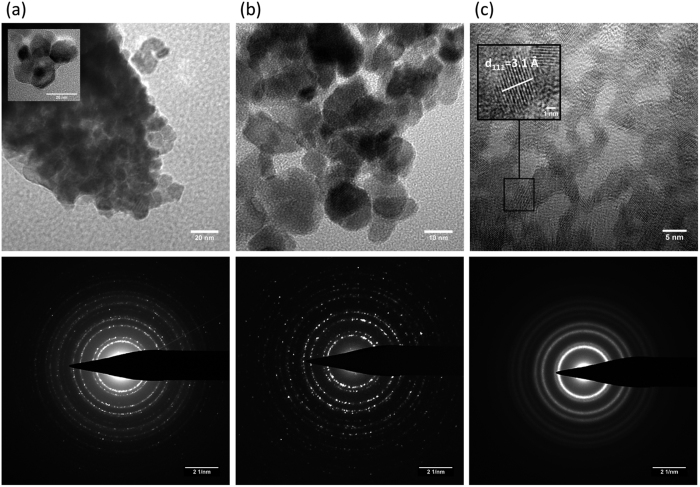
HRTEM images and electron diffraction patterns of studied Pu NPs. **(a)** nanostructured PuO_2_, **(b)** sonochemical Pu colloid, **(c)** hydrolytic Pu colloid. Inset of Fig. 3a shows sintered PuO_2_ nanoparticles. Samples were measured after 1 month of sampling.

Figure 2a reveals that PuO_2_ obtained at 485 °C and used for Pu colloid preparation has a nanostructured morphology composed of 10–11 nm sintered particles. Similar nanograined architectures have been recently observed for UO_2_ and ThO_2_ oxides obtained by the calcination of U(IV) and Th(IV) oxalates at 600 °C^24^. A corresponding morphology can also be observed in a low resolution TEM image of ThO_2_ employed in this work (Figure S3). Sonochemical and hydrolytic Pu colloids shown in Fig. 2b,c are composed of NPs with an average size of 7 and 3 nm, respectively, and are smaller than those of nanostructured PuO_2_ (Fig. 2a). Sintered grain boundaries evidenced in the nanostructured PuO_2_ (Fig. 2a) are not observed for sonochemical Pu colloid. Additional HRTEM images of these materials are provided in Figure S4. The larger particle size of sonochemical colloid in comparison to hydrolytic colloid is in good agreement with their UV-Vis-NIR spectra (Fig. 1b). The electron diffraction analysis confirmed the *fcc* crystal structure (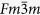 space group) for nanostructured PuO_2_ and for both colloids as well. The HRTEM data are summarized in Table 1. The abnormally low value of the d_111_-spacing for the hydrolytic Pu colloid results from the diffuse electron diffraction rings that leads to significant uncertainty in the d-spacing determination. In this case, the direct measurement of d_111_-spacing from HRTEM images provides better correlation with the expected 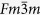 symmetry for this colloid. It should be emphasized that the intrinsic Pu colloid obtained by PuO_2_ sonolysis in the presence of pure argon also yields PuO_2_ NPs which were found to be similar to those obtained with 10%CO/Ar gas mixture. The particle size of hydrolytic colloid studied in this work matches well with that reported for Pu intrinsic colloids prepared by basic hydrolysis of Pu(IV)^7^,^25^. On the other hand, the autoxidation of Pu(III) in weakly acid solutions yields larger colloidal particles^25^ which is consistent with the proposed redox mechanism of sonochemical Pu colloid formation.

The study of colloid aging was not a primary objective of this work. Nonetheless, it can be mentioned that the HRTEM images of hydrolytic colloidal particles shown in Fig. 2b,c and Figure S4 do not reveal any noticeable variation in colloidal particle size or morphology after 6 months of storage. Similarly, no variation in their Vis-NIR spectra and pH values was observed even after one year aging (Figure S5). In the literature, high stability of hydrolytic Pu colloids towards sedimentation is usually attributed to a small particle size and to the stabilizing role of adsorbed anions originated from the initial acidic Pu(IV) solution^1^,^4^. By contrast, some precipitate is observed in the sonochemical colloidal solution after approximately one year aging. This process is accompanied by slight pH decrease from 3.2 to 2.8 which is probably related to the oxolation of Pu(IV) hydroxo species leading to the drop of the particle charge. The Vis-NIR spectra of the sonochemical colloid are stable for at least 3 months (Figure S5). However, the study of sonochemical colloid metastability needs further investigation.

### Pu L_III_-edge X-ray absoption spectroscopy (XAS) study

**Figure 3:**
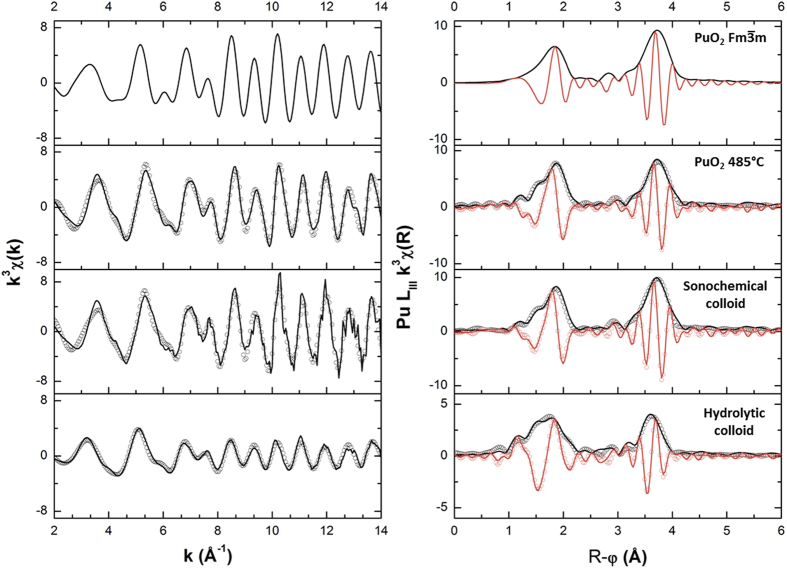
Experimental k^3^-weighted EXAFS spectra (left column) and corresponding real parts obtained for Fourier Transform magnitudes (right column). Data are obtained in the 2 Å^−1^ < k < 14 Å^−1^ range. Spectra were obtained after 1 month of sampling.

X-ray absorption near-edge structure (XANES) spectra shown in Figure S6 exhibit a white line at 18067.2 ± 0.5 eV that unequivocally indicates a Pu(IV) oxidation state for all the samples studied. Figure 3 shows the experimental k^3^-weighted extended X-ray absorption fine structure (EXAFS) functions and the corresponding k^3^-weighted Fourier transforms with real parts (2 Å^−1^ < k < 14 Å^−1^). A simulated EXAFS spectrum of 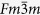 PuO_2_ is shown for comparison. For all the k^3^-weighted EXAFS spectra two types of oscillations are observed: lower frequency oscillations corresponding to Pu-O shell at low k values (2–8 Å^−1^), and higher frequency oscillations corresponding to longer Pu-Pu interactions at high k values (8–16 Å^−1^) in agreement with recently reported data for PuO_2_^26^,^27^,^28^. EXAFS spectra for all of the studied samples exhibit slightly lower amplitudes than those of simulated PuO_2_. This has been previously interpreted in terms of distortions in the first Pu(IV) coordination sphere leading to more than one Pu-O distance^28^.

In this work, the successful fit of EXAFS spectra for nanostructured PuO_2_ and Pu intrinsic colloids has been achieved using a triple oxygen shell model for the first coordination sphere of Pu(IV) as proposed by Soderholm *et al*.^9^ to describe the structure of small (~1 nm) Pu clusters. According to this model, Pu-O distances composing the first coordination sphere were subdivided into 3 groups: short (1.93–2.23 Å, O_s_), medium (2.23–2.63 Å, O_m_), and long (2.63–3.13 Å, O_l_). Furthermore, 15 metrical parameters were defined for the fit using Arthemis software: R_i_, N_i_, σ^2^_i_, i = O_s_-O_m_-O_l_-Pu-O2, where Pu and O2 indicate Pu-Pu and Pu-O interactions in the second coordination sphere. The first step of EXAFS data analysis was an evaluation of the experimental spectra in comparison to simulated spectrum of 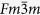 PuO_2_. Metrical parameters were first restricted to reported values for crystalline PuO_2_^25^: S_0_^2^ = 0.9, R_Om_ = 2.337 Å, R_Pu_ = 3.816 Å, R_O2_ = 4.472 Å, N_Om_ = 8, N_Pu_ = 12, N_O2_ = 24, N_Os_ = N_Ol_ = 0 and σ^2^_O2_ = 0.008 Å^2^; whereas ΔE_0_, σ^2^_Om_ and σ^2^_Pu_ were the only floating parameters. The given σ^2^ errors represent the standard deviation of experimental parameters from the reference PuO_2_. The second step consisted of improving the fit to determine the colloid fine structure by allowing all metrical parameters (R_i_, N_i_, σ^2^_i_) to float. The amplitude reduction factor (S_0_^2^) was fixed at 0.9 as proposed by Martin *et al*.^25^ and Rothe *et al*.^27^ Lastly, the sum of the coordinated oxygen atoms N_Os_, N_Om_ and N_Ol_ was restricted to a range of 6–10 to maintain a reasonable coordination number for Pu(IV).

Table 2 summarizes the metrical parameters obtained from the samples studied using three oxygen shell fitting procedure. These results show that Pu-Pu distance for nanostructured PuO_2_ and for both colloids is very close to that of reference PuO_2_. However, the number of Pu-Pu (N_Pu_) and Pu-O interactions in the second coordination sphere (N_O2_) are decreased with the decreasing particle size as measured by HRTEM (Table 1). In general, this trend is also observed for the number of Pu-O bonds in the first shell (N_Om_). However, sonochemical colloid exhibits larger relative Pu-Pu FT magnitude (Fig. 3) and almost approach the same value of N_Om_ compared to nanostructured PuO_2_ indicating an improved local ordering of colloidal plutonium NPs obtained via sonochemistry. The very low N_Pu_ value for the hydrolytic colloid can also be ascribed to the fact that the Pu-Pu distances at the particle shell interface result in a variety of deviations with similar but not identical Pu-Pu distances. The individual EXAFS scattering waves can undergo destructive interference which results in a dramatic decrease of the related coordination number^29^.

**Figure 4:**
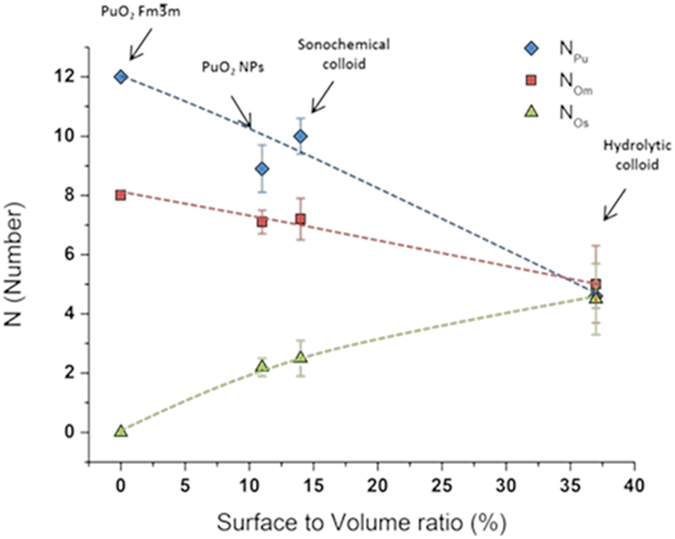
Dependence of N_Om_ (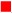), N_Pu_ (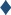), and N_Os_ (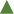) on plutonium particle surface-to-volume ratio. The results for nanostructured PuO_2_, sonochemical, and hydrolytic Pu intrinsic colloids are compared with 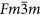 PuO_2_ (S/V = 0).

In contrast to the medium Pu-O bonds, the number of short Pu-O bonds (N_Os_) progressively increases with the decrease in Pu particle size. This trend is clearly evidenced from Fig. 4 that shows the dependence of N_Pu_, N_Om_ and N_Os_ on the particle surface-to-volume atom ratio, S/V. The S/V values have been calculated using Crystal Maker software to establish the minimum number of Pu or O atoms required for uniform coating of spherical PuO_2_ nanoparticle. In the literature, short Pu-O bonds at about 2.2 Å were attributed to terminal Pu-OH moieties and/or to the μ^3^-O atoms that reside on the edge of the particle and bonded to three Pu(IV) ions, medium bonds at about 2.34 Å to bulk plutonium dioxide, and longer bonds at ~2.7–3.0 Å to coordinated water molecules^9^,^28^. Consequently, the plot in Fig. 4 confirms the relatively higher contribution of hydrolysed Pu(IV) moieties for smaller PuO_2_ NPs. Taking into account that the isoelectric point of intrinsic Pu(IV) colloids is at pH = 8.6^6^, the stability of sonochemical Pu colloid toward sedimentation in the absence of stabilising agents can be assigned to the surface protonation of Pu-O and Pu-O-Pu bonds. It is worthwhile to mention that in contrast to sonochemical Pu colloid, the EXAFS spectra of nanostructured PuO_2_ do not show the presence of coordinated water which can be attributed to the fact that this species was examined as a solid sample. Both HRTEM and EXAFS data indicate that Pu NPs of both colloids are composed of a quasi-stoichiometric PuO_2_ core and a hydrolyzed Pu(IV) shell. In these terms, the distortion of Pu-O bonds observed for PuO_2_ NPs can be interpreted mostly as resulting from a particle size effect. The EXAFS data for nanostructured PuO_2_ and sonochemical colloid, however, suggest that some local disordering in the first coordination sphere of Pu(IV) is also possible.

### Study of the oxygen K-edge near-edge X-ray absorption spectroscopy (NEXAFS) with a Scanning Transmission X-ray Microscope (STXM)

The normal contrast X-ray images and STXM elemental maps (from the O K edge and Pu M_5_ edges) are shown in Figures S7 and S8. Figure S7 reveals that sonochemical and hydrolytic Pu colloids exhibit different drying behavior on Si_3_N_4_ windows which were used for the STXM NEXAFS measurements. After drying, the aggregates of sonochemical Pu colloid are randomly distributed along concentric circles whereas dried hydrolytic Pu colloid forms a dense and homogeneous slick at the edge of the drop front. In addition, sonochemical colloids flocculate to form ~100 nm irregular agglomerates of smaller particles which cannot be fully resolved by the STXM technique. For this sonochemical sample, oxygen and plutonium maps are highly correlated with one another and the STXM resolution limits, indicating that oxygen atoms are mostly associated with plutonium ones. Contrasting to this behaviour, the drying of hydrolytic Pu colloid yields very diffuse, irregular packed solid architectures which are made up of much smaller particle size building blocks compared to the sonochemical colloid. Furthermore, the hydrolytic colloid oxygen map exhibits a larger surface area compared to that of its respective plutonium map. This observation suggests that the small colloidal particles of plutonium are surrounded by a plutonium-free oxygen species likely composed of sodium nitrate.

**Figure 5:**
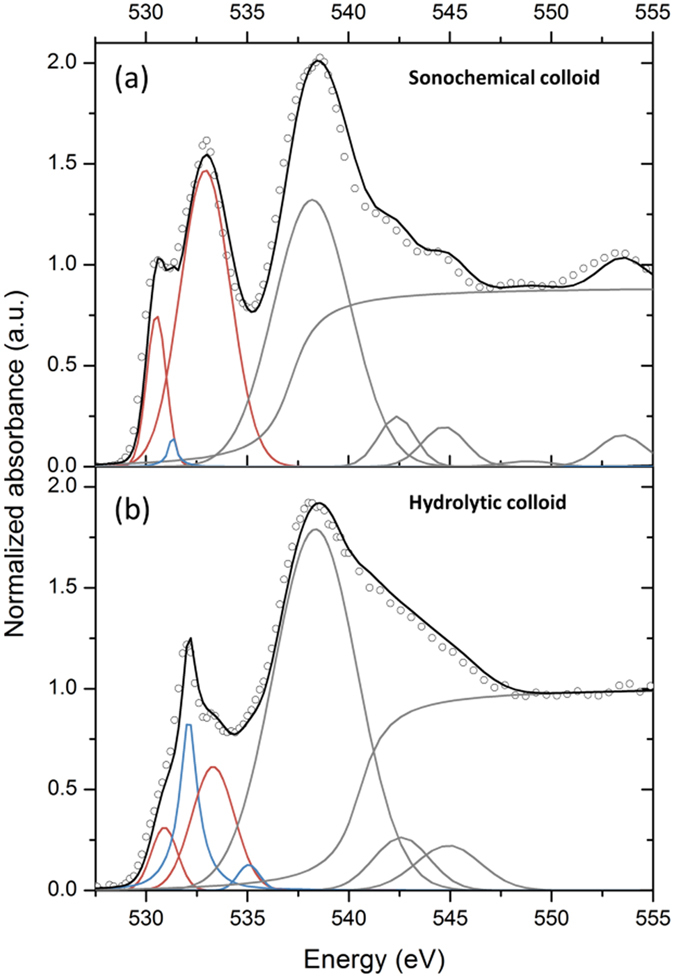
Experimental O K-edge XAS spectra (open circle) and their fitting curves (black line). **(a)** sonochemical and **(b)** hydrolytic Pu colloids. The individual Gaussian and arctangent contributions to the model are shown as red, blue and grey lines respectively. Spectra were obtained after 2 months of sampling.

The oxygen K-edge NEXAFS spectra measured close to the drop edge (Figure S7a,b, 0 μm arrow) are shown in Fig. 5 for both colloids. The background subtraction and spectral normalization have been performed according to previously reported procedures^30^. The spectra were evaluated by curve fitting analysis using constrained Gaussian line shapes and an arctan step function. Gaussian functions were constrained to reproduce the pre-edge, edge and post-edge of crystalline PuO_2_ as reported in the literature^30^,^31^,^32^.

Two pre-edge peaks in the spectrum of sonochemical Pu colloid at 530.5 eV (O *2p*–Pu *5f*) and 532.9 eV (O *2p*–Pu *6d*) are similar to those of reference PuO_2_ (Figure S9)^32^. The main edge peak centred at 538.2 eV and the post-edge contributions in the 542–568 eV range are also consistent with the values reported for PuO_2_^32^. Contrasting to the sonochemical colloid, the O K-edge NEXAFS spectrum from the hydrolytic Pu colloid resembles PuO_2_ to a much less extent. The intensity of pre-edge peaks at 530.5 eV and 532.9 eV are significantly lower than those of the sonochemical colloid. Moreover, the spectrum of hydrolytic colloid does not exhibit multiple scattering features at 555 eV and 568 eV that are typical for crystalline PuO_2_. The main edge shape itself is split in two peaks, one at 538 eV and the other at 543 eV, that can be attributed to the strong influence of hydrated and/or hydrolysed forms of Pu(IV) on the total oxygen state^31^,^32^. In addition, the contribution from nitrate ions to the experimental O K-edge NEXAFS spectra of hydrolytic Pu colloid also cannot be completely excluded^33^. It can be concluded that the difference between the two studied colloids is the stronger influence of hydrolysed Pu(IV) moieties at the surface shell of the smaller colloidal nanoparticles (hydrolytic). According to Fig. 4, the fraction of surface Pu(IV) ions is about 36% for hydrolytic colloid and only 14% for sonochemical colloid. Consequently, the O K-edge NEXAFS spectra of hydrolytic colloid reflects the hydrolysed species to greater extent than those of sonochemical colloid and is in a good agreement with Pu L_III_ edge EXAFS spectra.

## Methods

### PuO_2_ and ThO_2_ samples preparation

Experiments with plutonium were performed at Atalante Facility of Marcoule Research Centre, France. The stock solution of a “^239^Pu” isotope mixture (96.9% ^239^Pu, 2.98% ^240^Pu, 0.04% ^241^Pu, 0.06% ^242^Pu) was purified by a standard anion-exchange method. The specific alpha-activity of the “^239^Pu” sample was found to be equal to 4.0·10^6^ Bq·mg^−1^. Plutonium oxide samples were obtained by conventional Pu(IV) oxalate calcination under air at 485, 520 and 600 °C to obtain PuO_2_ with different specific surface areas. Powder X-ray diffraction (XRD) analysis showed the characteristic patterns of the fluorite structure (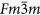) typical for PuO_2_ (ICSD #186183) for all of the calcined samples. For comparison, some experiments were performed with ThO_2_ which has been prepared by calcination of Th(IV) oxalate in the presence of air at 485 °C. The specific surface areas for actinide oxides used in the experiments are summarized in Table S2. All solutions were prepared with milli-Q water (18.2 MΩ·cm at 25 °C). Commercially available reagents of the best purity were used as received without further purification.

### Sonochemical experiments

In a typical sonochemical experiment, 200 mg of PuO_2_ powder suspended in 50 mL of water was treated with 20 kHz ultrasound in a thermostatically controlled closed batch reactor using a 1 cm^2^ irradiating surface area Ti6Al4V titanium alloy probe powered by a 750 W Sonics generator. The probe was immersed reproducibly below the surface of the sonicated suspension. The acoustic power density, P_ac_ (W·mL^−1^), transmitted to the liquid medium, was measured using a conventional thermal probe method and was equal to 0.34 W·mL^−1^. The sonoreactor was installed inside a glovebox for manipulation with radioactive materials. For all of the experiments, the suspensions were sparged with the desired gas (Ar, 10% CO/Ar or 20% O_2_/Ar) for about 30 min before sonication and during the ultrasonic treatment at a rate of 100 mL·min^−1^. A temperature of 20–21 °C inside the sonoreactor was maintained during sonolysis by a thermocryostat Lauda Ecoline RE 210 connected from outside the enclosure. After sonication, the suspension was centrifuged twice at 9000 rpm for 15 min. to separate large non-colloidal particles of PuO_2_ from the Pu colloid.

Hydrolytic Pu colloids were used as references in some experiments. These colloids were obtained by dilution of a 0.459 M Pu(IV) solution containing 2 M of HNO_3_ in pure water. The absence of Pu(VI) in this solution was controlled by UV-Vis spectroscopy. Typically, a calculated aliquot of Pu(IV) solution was injected into 10 mL of pure water under conditions of vigorous stirring. The pH value of obtained colloidal suspensions was found to be equal to 2.8.

### Spectroscopic, XRD, specific surface area, and microscopic characterisation

UV-vis-NIR absorption spectra of Pu colloids were recorded using a Shimadzu UV3600 spectrophotometer with a 1 cm quartz cell. The concentration of Pu in colloidal solutions was determined by alpha spectroscopy (7401 Canberra). XRD patterns of the metal oxide samples were acquired on a Bruker D8 Advance Diffractometer equipped with a Cu K_α_ (λ = 1.54056 Å) radiation source and a Lynx Eye detector. Solid samples were immobilized in epoxy resin to prevent the dispersal of harmful radioactive powder. The specific surface area of metal oxides was acquired by N_2_ adsorption/desorption isotherms at 77 K using a Micromeritics Gemini 2360 apparatus. The samples were degassed under vacuum at 393 K overnight prior to analysis. The isotherm data were fitted using the Brunauer-Emmet-Teller (BET) model to calculate the surface area values. The scanning electron microscopy (SEM) images were obtained with a Quanta 200 ESEM FEG (ThO_2_) and a Zeiss Supra 55 (PuO_2_) devices. High-resolution transmission electron microscopy (HRTEM) measurements were performed at the Joint Research Centre of the Institute for Transuranium Elements, Karlsruhe, Germany (JRC-ITU) using a TecnaiG2 (FEI^TM^) 200 kV microscope equipped with a field emission gun, modified during its assembly to enable the examination of radioactive samples. XAFS measurements were performed at the European Synchrotron Radiation Facility (ESRF) at the Rossendorf Beamline (BM20-ROBL) using a Si(111) double crystal monochromator. BM20 is equipped with a collimating and focusing mirror, and the beam spot size is reduced by horizontal and vertical slits to 3 × 1 mm. Spectra were recorded in transmission mode with 3 argon-filled ionization chambers giving I_0_, I_1_ and I_2_. The energy was calibrated *in situ* by measuring after the sample (I_1_/I_2_) the first derivative XANES spectrum of a Zr foil defined at 17988 eV. Experimental spectra were recorded at Pu L_III_ edge and the ionization energy (E_0_) was preliminary defined at the maximum of the white line at 18068 eV and varied as free fit parameter. The energy range investigated for EXAFS structure determination was chosen depending on Pu content in the sample from 2 Å^−1^ to 14–17 Å^−1^. EXAFS data analysis was performed with Athena and Arthemis software from the IFEFFIT package^34^. Further efforts were made to optimize the atomic background function to reduce the amplitude between 0 and 1.2 Å on the Fourier transformed magnitude (FT). Back scattering amplitudes and phases functions were obtained from FEFF8.4^35^ on the basis of the 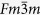 fluorite structure of PuO_2_ (lattice parameter 5,396 Å, ICSD #186183). Soft X-ray measurements were performed at the Molecular Environmental Science (MES) Beamline 11.0.2 of the Advanced Light Source (Lawrence Berkeley National Laboratory, USA) equipped with a scanning transmission X-ray microscope (STXM)^30^. The available energy range was approximately 100–2000 eV and the energy resolution of the measurements was less than 0.1 eV at the oxygen K edge. Transmitted X-rays were measured as a function of the sample position (synchronized by an interferometer) to obtain X-ray images by optical density contrast. Image resolution was determined by the spot size (~25 nm in this study) and position steps. Element map and NEXAFS spectra were obtained using image sequence scans over different photon energy ranges (520 to 600 eV for O K edge, 750 to 890 for Pu N_4,5_ edges) using the STAKS procedure implemented in the aXis 2000 software^30^.

## Additional Information

**How to cite this article:** Dalodière, E. *et al*. Insights into the sonochemical synthesis and properties of salt-free intrinsic plutonium colloids. *Sci. Rep.* **7**, 43514; doi: 10.1038/srep43514 (2017).

**Publisher's note:** Springer Nature remains neutral with regard to jurisdictional claims in published maps and institutional affiliations.

## Supplementary Material

Supplementary Information

## Figures and Tables

**Table 1:** HRTEM analysis of nanostructured PuO_2_ and Pu intrinsic colloids. The d-spacing for plutonium species were obtained from electron diffraction data.

	Particle size (nm)*	d_111_ (Å)	d_200_ (Å)	d_220_(Å)
Nanostructured PuO_2_	10.8 ± 1.7	3.18	2.76	1.94
Sonochemical colloid	7.1 ± 1.2	3.12	2.65	1.88
Hydrolytic colloid	2.9 ± 0.4	3.03	2.63	1.84
		3.10**		
PuO_2_ ( 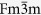 )***		3.12	2.70	1.91

*Average value from at least 50 parallel measurements. **Measured from HRTEM images. ***ICSD #186183.

**Table 2:** Metric parameters calculated from k^3^-weighted EXAFS spectra using a 3O-shell fitting procedure in comparison to simulated 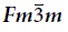 PuO_2_ oxide.

Sample	Shell	N	(ΔN)	σ^2^ (Å^2^)	(Δσ^2^)	R(Å)	(ΔR)
Simulated PuO_2_							
	O_s_	0.0	0.0	—	—	—	—
	O_m_	8.0	0.0	—	—	2.34	0.01
	O_l_	0.0	0.0	—	—	—	—
	Pu	12.0	0.0	—	—	3.81	0.01
	O2	24.0	0.0	—	—	4.47	0.01
PuO_2_ 485 °C							
	O_s_	2.2	0.3	0.005	0.002	2.19	0.01
	O_m_	7.1	0.4	0.005	0.001	2.33	0.01
	O_l_	0	—	—	—	—	—
	Pu	8.9	0.8	0.005	0.001	3.81	0.01
	O2	13.9	3.3	0.007	0.002	4.45	0.02
Sonochemical colloid							
	O_s_	2.5	0.6	0.004	0.003	2.17	0.02
	O_m_	7.2	0.7	0.004	0.001	2.32	0.01
	O_l_	0.3	1.0	0.010*	—	2.76	0.20
	Pu	10.0	0.6	0.004	0.010	3.80	0.01
	O2	9.5	1.7	0.010	0.002	4.44	0.03
Hydrolytic colloid							
	O_s_	4.5	1.2	0.007	0.003	2.24	0.02
	O_m_	5.0	1.3	0.007	0.003	2.39	0.02
	O_l_	0.5	0.7	0.010*	—	3.02	0.25
	Pu	4.6	0.4	0.005	0.002	3.80	0.01
	O2	2.5	5.7	0.010*	—	4.47	0.25

0.5% < R-factor < 3%, 0.5 eV < ΔE_0_ < 2 eV, S_0_^2^ = 0.9. *Fixed values.
