# Supplementary material for: Insights into the sonochemical synthesis and properties of salt-free intrinsic plutonium colloids
Source: Sci Rep. 2017 Mar 3;7:43514. doi: 10.1038/srep43514 (PMC5335258; doi:10.1038/srep43514)
Supplement: Supplementary Information [file srep43514-s1.pdf]

Supplementary Information

Insights into the sonochemical synthesis and properties of salt-free intrinsic plutonium colloids

Elodie Dalodière, Matthieu Virot, Vincent Morosini, Tony Chave, Thomas Dumas, Christoph Hennig, Thierry Wiss, Oliver Dieste Blanco, David K. Shuh, Tolek Tyliszczak, Laurent Venault, Philippe Moisy & Sergey I. Nikitenko

(email: serguei.nikitenko@cea.fr)

Sample preparation and analysis

Analysis of thorium in sonicated solution. The thorium concentration was determined quantitatively using inductively coupled plasma optical emission spectroscopy (ICP-OES) with a Spectro Arcos apparatus (Spectro Analytical Instruments GmbH) equipped for axial plasma observation. After removal of solid particles by centrifugation (twice at 9000 rpm for 15 min), the samples were diluted using 0.3 M HNO_3. The concentration of the element was evaluated using calibration curves obtained with certified Th standard solution (SCP Science). The detection limit of Th was about 0.1 ppm.

pH measurements. The pH values were measured after the separation of non-colloidal particles using Cyberscan pH300 meter equipped with HACH glass electrode calibrated against standard pH buffers (pH 1.68 – 10.00, Merck).

HRTEM and SEM sample preparation. One drop of non-diluted colloid was deposited on carbon coated copper grid and dried on air prior analysis. Plutonium oxide sample was dispersed in water and then one drop of suspension was deposited on the grid as for colloids. Samples for SEM analysis were prepared by similar procedure using a standard pin type mount holder.

EXAFS sample preparation. 200 μL of colloidal solution were placed in a sealed plastic cell developed for BM-20-ROBL Beamline at ESRF, Grenoble. Solid samples of PuO_2 were prepared by the mixing of 5 mg PuO_2 powder with BN powder, pressed in pellets and then sealed in a plastic holder. All samples of the sonochemical colloid were obtained after non-colloidal particles removal by centrifugation.

Kinetics of Pu(IV) disproportionation

The rate of Pu(IV) disproportionation is described by the second-order kinetic equation in $Pu(OH)^{3+}$ concentration according to Rabidau S.W. et al. Proc. 2nd UN Int. Conf. on Peaceful Uses of At. Energy, 1958, 28, 361-372, Geneva, Switzerland:

$$-d[Pu(IV)]/dt = 2(k_1[H^+]^{-3} + k_2[H^+]^{-4})[Pu(OH)^{3+}]^2$$

where $[Pu(OH)^{3+}] = [Pu(IV)](1 + pK_1[H^+]^{-1})^{-1}$ and K_1 is a first hydrolysis constant of Pu(IV). This kinetic equation indicates that Pu(IV) hydrolysis is required for effective Pu(IV) disproportionation. Consequently, at sufficiently high Pu(IV) concentration formation of hydrolytic colloid should be accompanied by Pu(IV) disproportionation.

SEM images of PuO_2 and ThO_2 before and after 30 h of sonolysis in water

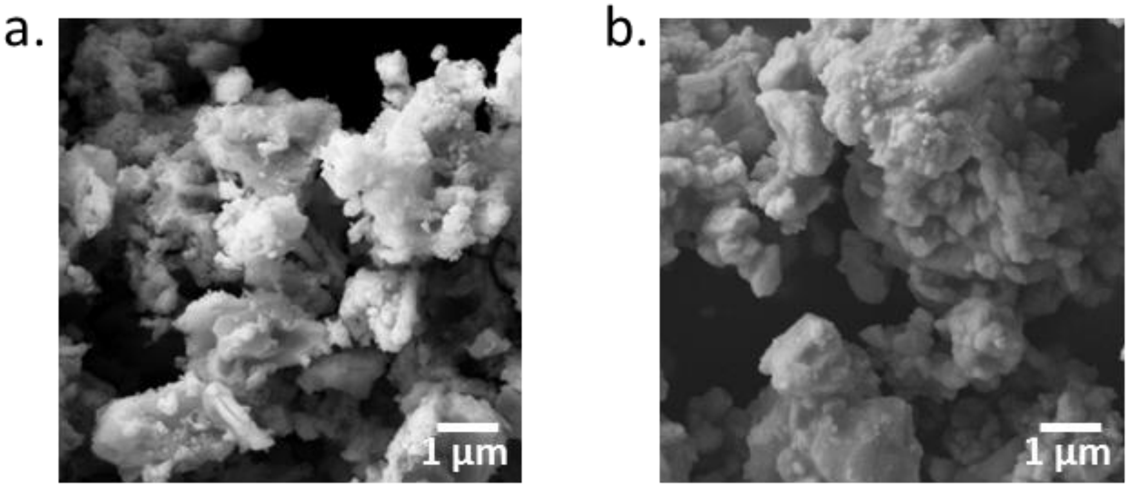

Figure S1. a. PuO_2 obtained by calcination of Pu(IV) oxalate at 485°C before ultrasonic treatment, **b.** PuO_2 after 30 h of sonolysis in water in the presence of 10%CO/Ar gas mixture (f= 20 kHz, P_{ac}= 0.34 W mL^{-1}, T= 20°C).

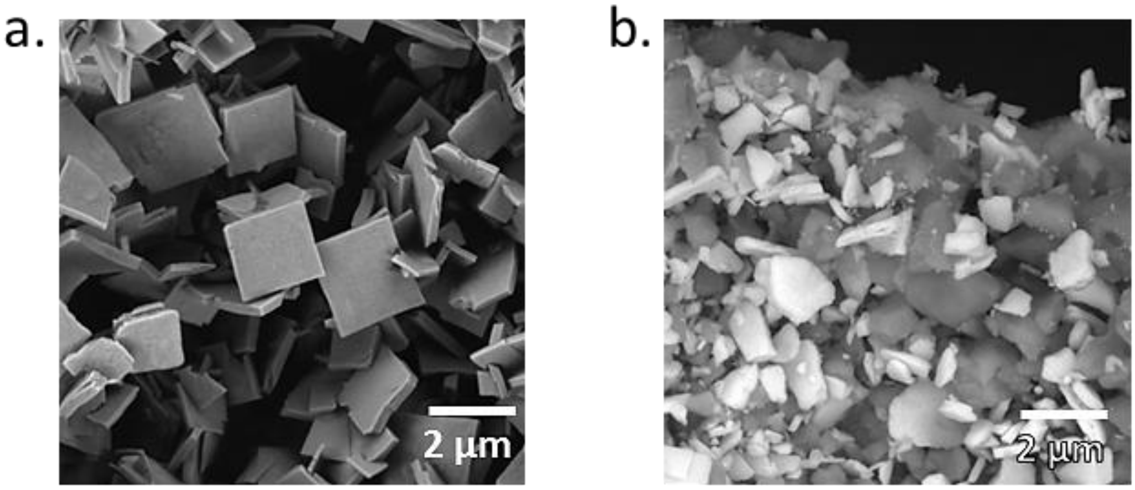

Figure S2. **a.** ThO_2 obtained by calcination of Th(IV) oxalate at 485°C before ultrasonic treatment, and **b.** after 30 h of sonolysis in the presence of Ar.

Low- and high resolution TEM images of ThO_2 and PuO_2 samples

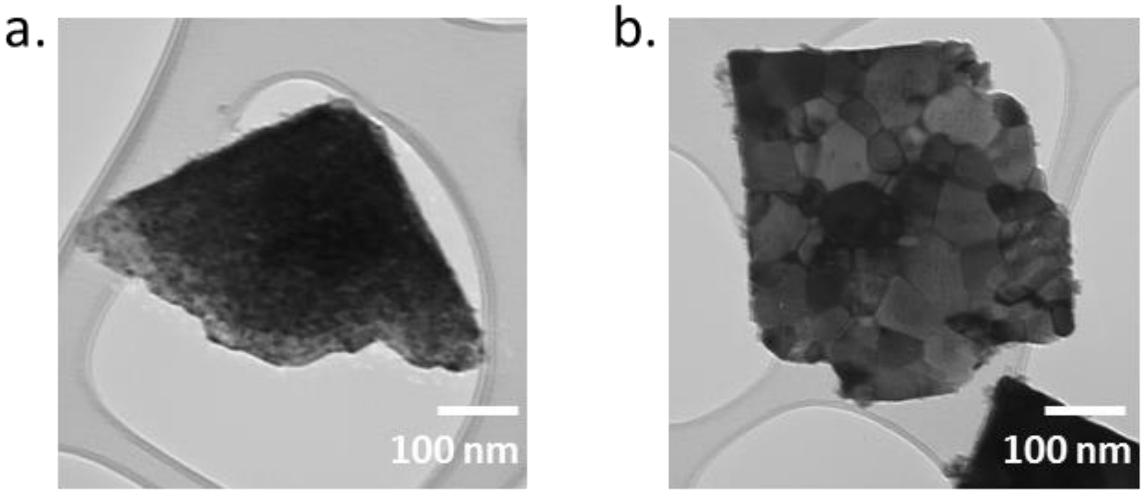

Figure S3. Low resolution TEM of ThO_2 prepared by calcination of Th(IV) oxalate at **a**. 485°C and **b**. 1000°C. The grain size is ~5-10 nm for powders heated at 485°C and ~30-70 nm heated at 1000°C. Measurements were made using a Jeol 1200 EXII microscope (120 eV).

Table S1. Yield of sonochemical colloid obtained after 30 h of sonolysis (f= 20 kHz, P_{ac}= 0.34 $W \cdot mL^{-1}$, 200 mg of metal oxide powder in 50 mL of water) of nanostructured PuO_2 (S_{BET}= 20 $m^2 \cdot g^{-1}$) and ThO_2 (S_{BET}= 17 $m^2 \cdot g^{-1}$) aqueous suspension as a function of saturating gas. **G** values were measured after the removal of non-colloidal AnO_2 particles by alpha spectroscopy for Pu and by ICP-OES for Th. Concentration of Th was less than the detection limit (0.1 ppm).

	**Bubbling gas**	**G (µmol)**
PuO_2 (20 $m^2.g^{-1}$)		
	Ar	34
	10%CO/Ar	85
	20%O_2/Ar	0
ThO_2 (17 $m^2.g^1$)		
	Ar	0

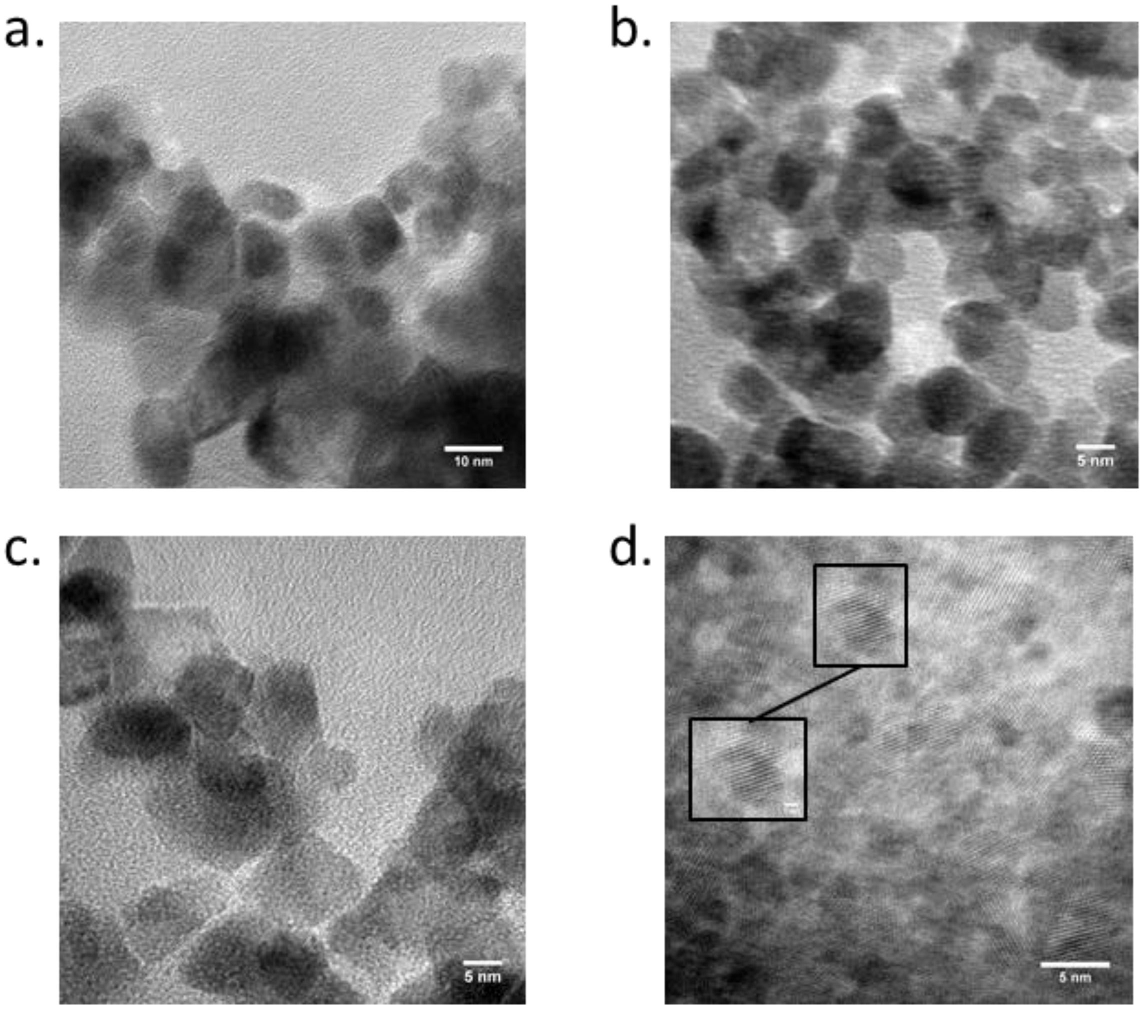

Figure S4. Additional HRTEM images of **a.** nanostructured PuO_2 obtained at 485°C, **b.** sonochemical Pu colloid obtained in the presence of 10%CO/Ar, **c.** sonochemical colloid obtained in the presence of pure argon, and **d.** hydrolytic colloid aged 6 months.

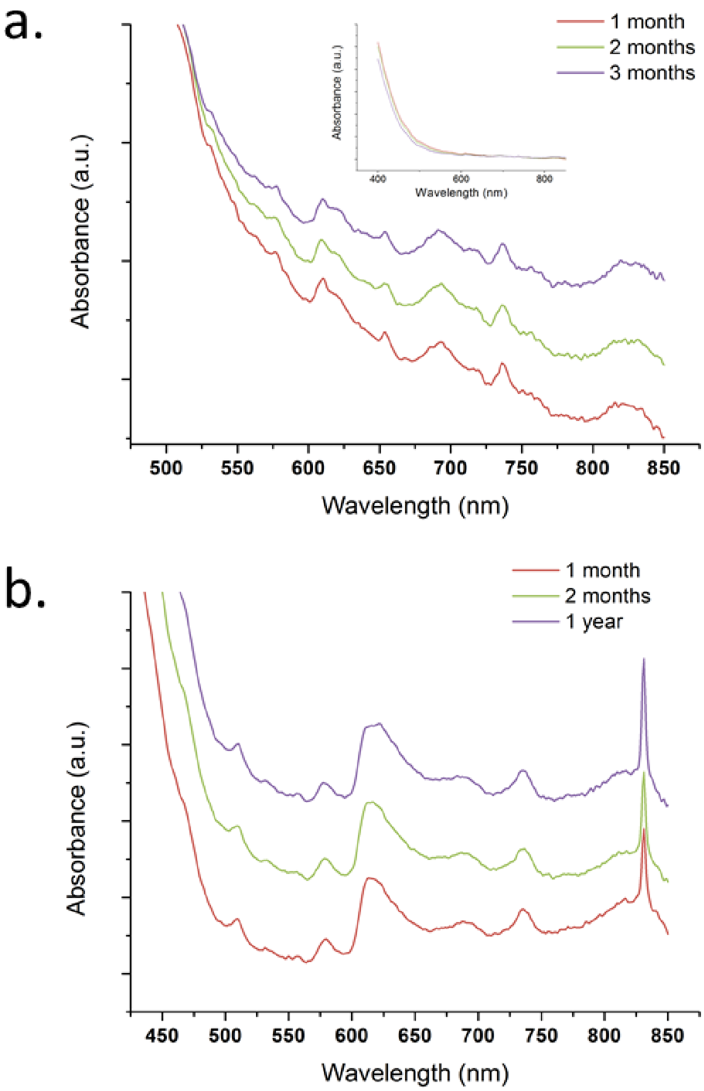

Figure S5. Vis-NIR spectra of sonochemical, [Pu]= $1.7 \cdot 10^{-3}$ M, pH= 3.2 (a) and hydrolytic, [Pu]= $2.3 \cdot 10^{-3}$ M, pH= 2.8 (b) colloids collected during storage in closed glass flasks. The spectra were shifted for better visibility.

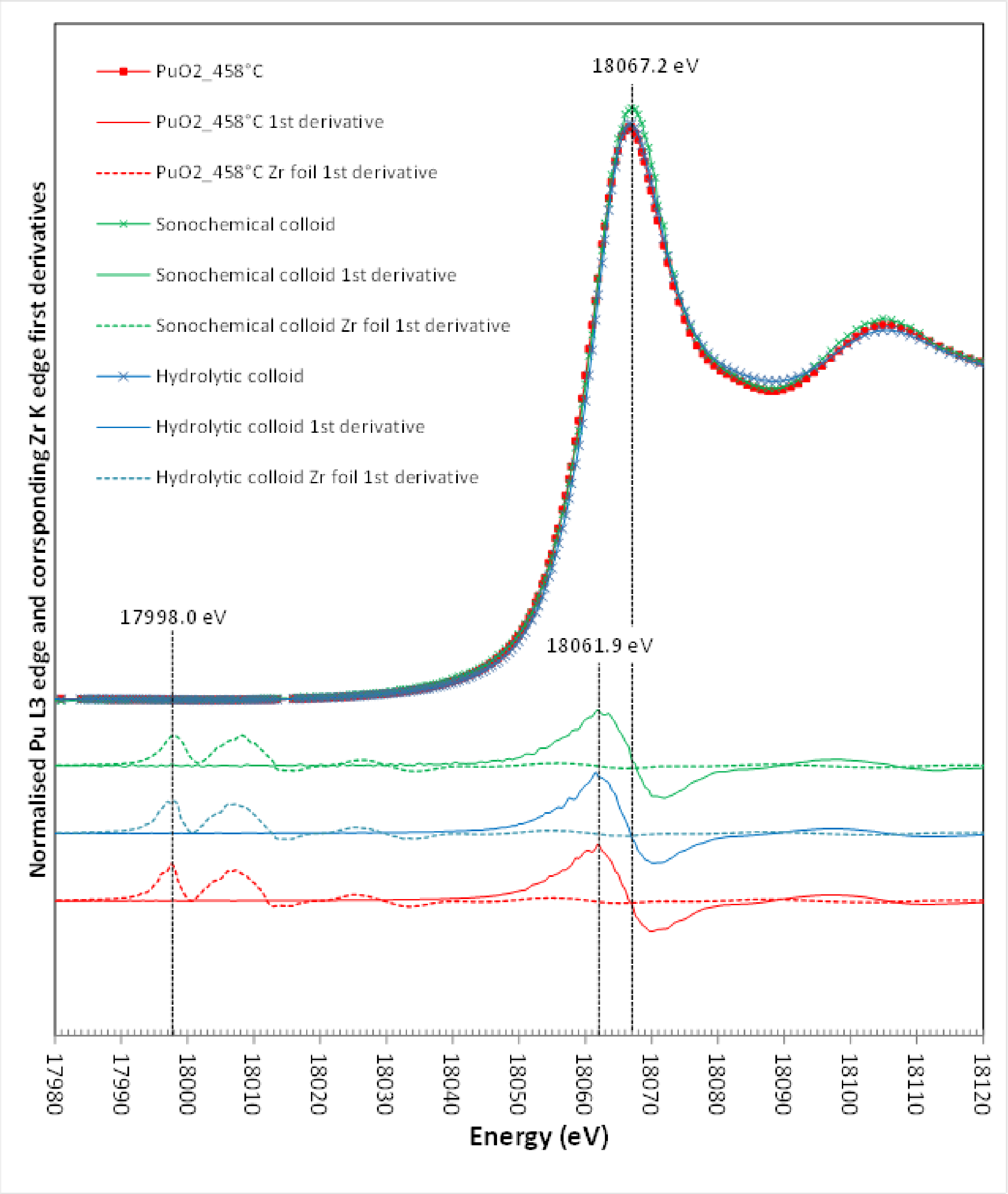

Figure S6. **XANES Spectra** of the nanostructured PuO_2 obtained at 485°C, sonochemical and hydrolytic colloids.

STXM and NEXAFS Measurements

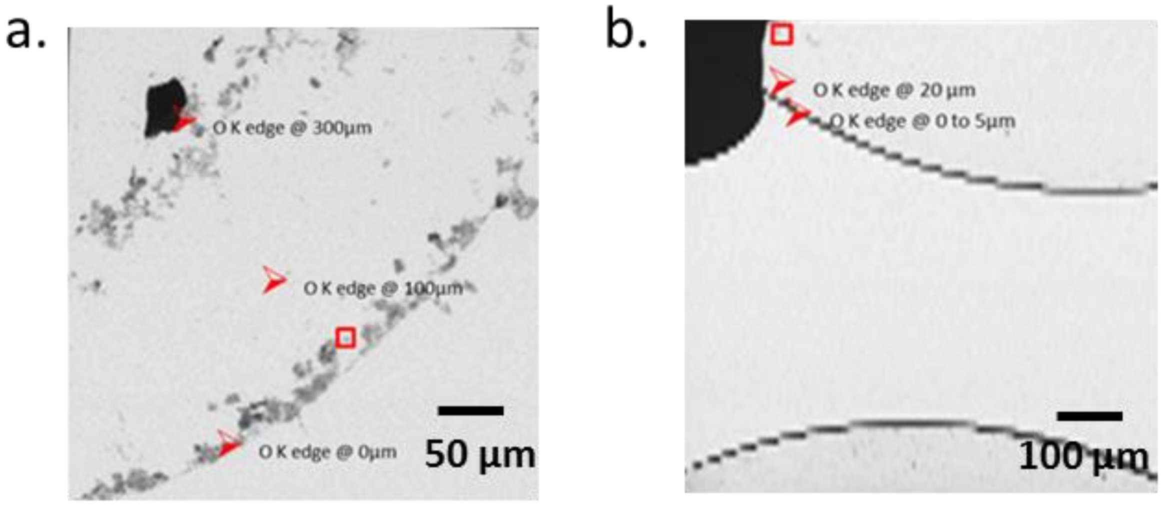

Figure S7. Normal contrast X-ray images of the STXM Si_3N_4 windows for **a.** dried sonochemical colloid and **b.** hydrolytic plutonium colloids. Red squares indicates position for STXM images presented. Red arrows indicate position for O K edge NEXAFS analysis.

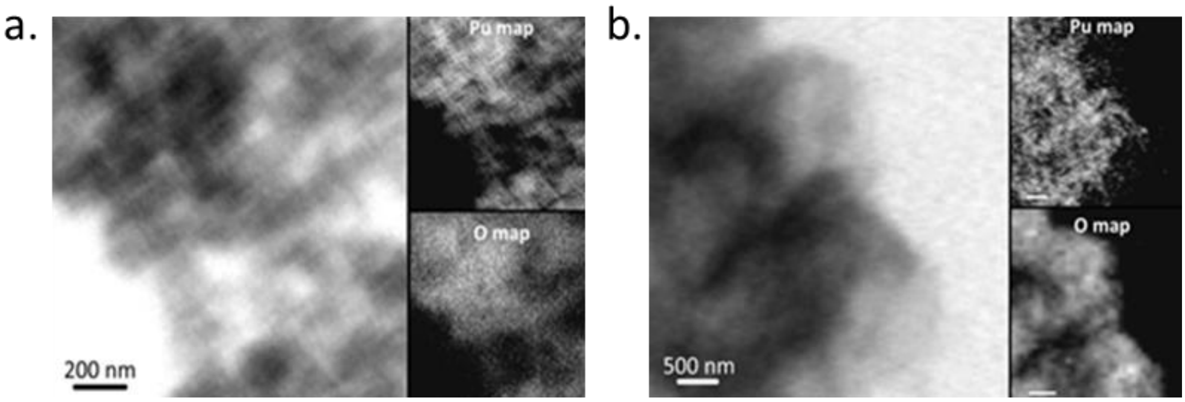

Figure S8. STXM images of dried plutonium colloids obtained at the maximum resolution for the ALS 11.0.2 beamline (25 nm) for **a.** hydrolytic plutonium colloid and **b.** sonochemical colloid. Plutonium maps were recorded between 780 eV and 800 eV and the O maps were obtained by contrast of the optical density at 525 eV and 540 eV. A 0.5 eV step line scan was used to determine the maximum absorption at both O K edge and Pu $M_{4,5}$ edges.

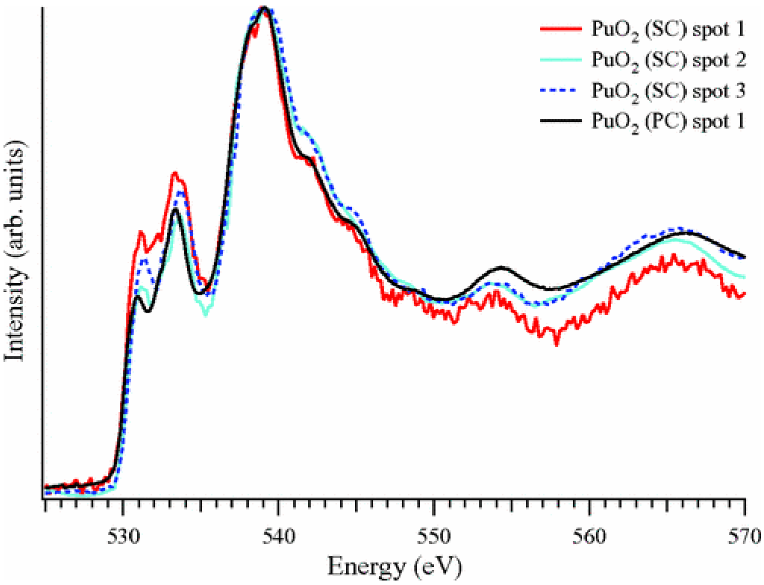

Figure S9. O K-edge XAS spectra collected for three areas/spots on the PuO_2 single crystal (SC) together with the spectrum of polycrystalline PuO_2. Spectra are normalized to the maximum and displayed on the incident photon energy scale. Reproduced with permission from the Ref. 31.

Table S2. Specific surface area (S_{BET}) of PuO_2 and ThO_2 samples.

**Temperature used for An(IV) oxalate calcination (°C)**		**S_{BET} ($m^2 \cdot g^{-1}$)**
Pu(IV)		
	485	20
	520	14
	600	3
Th(IV)		
	485	17
